# Acute lymphoblastic leukemia secondary to myeloproliferative neoplasms or after lenalidomide exposure

**DOI:** 10.1002/ccr3.1264

**Published:** 2017-12-06

**Authors:** Ahmad Alhuraiji, Kiran Naqvi, Yang O. Huh, Coty Ho, Srdan Verstovsek, Prithviraj Bose

**Affiliations:** ^1^ Department of Leukemia University of Texas MD Anderson Cancer Center Houston 77030 Texas; ^2^ Department of Hematopathology University of Texas MD Anderson Cancer Center Houston 77030 Texas; ^3^ Oklahoma Cancer Specialists and Research Institute Tulsa 74133 Oklahoma

**Keywords:** Acute lymphoblastic leukemia, *JAK2* V617F mutation, lenalidomide, myeloproliferative neoplasm

## Abstract

Philadelphia‐negative (Ph^−^) myeloproliferative neoplasms (MPN) do rarely transform to acute lymphoblastic leukemia (ALL). While causality is difficult to establish, a few cases of ALL arising after exposure to lenalidomide for registered indications (multiple myeloma, myelodysplastic syndrome with 5q deletion) have been described in the literature.

## Introduction

The classic Philadelphia chromosome‐negative (Ph^−^) myeloproliferative neoplasms (MPN), polycythemia vera (PV), essential thrombocythemia (ET), and primary myelofibrosis (PMF) share in common the activating Janus‐associated kinase 2 (*JAK2*) V617F mutation in approximately 95, 60, and 60% of cases, respectively 1, and universal activation of the *JAK*‐STAT (signal transducer and activator of transcription) pathway 2. The *JAK2* V617F mutation is specific to myeloid malignancies 3 and is not found in patients with “Ph‐like” acute lymphoblastic leukemia (ALL) who, however, have been shown to have other alterations involving *JAK2*, including both fusions and point mutations 4. Differences in mutant allele burden, STAT1 signaling, order of mutation acquisition, and clonal heterogeneity have been invoked as potential explanations of how the same driver mutation can result in substantially different clinicopathologic entities 1.

Both PV and ET may progress to myelofibrosis (post‐PV MF (PPV‐MF) or post‐ET MF (PET‐MF)) which, as well as PMF itself, may represent an “accelerated phase” in the spectrum of progression of MPN to acute myeloid leukemia (AML) 5, 6. Considerable evidence supports the accumulation of a variety of genetic lesions during leukemic transformation (LT) of MPNs 7. *JAK2* V617F‐induced genomic instability may lead ultimately to LT 5. However, *JAK2* V617F^+^ MPNs often transform to *JAK2*‐wild‐type AML 8; in this situation, the chronic and leukemic phases could either be clonally related, arising from a shared pre‐*JAK2* ^V617F^ clone, or clonally unrelated, reflecting transformation of independent stem cells 5.

The immunomodulatory agent lenalidomide is an analog of thalidomide that is approved for the treatment of multiple myeloma (MM) 9, 10, myelodysplastic syndrome (MDS) with 5q deletion 11, and mantle cell lymphoma 12. It is used most widely in MM, as part of two‐ 9, 10, 13 and three‐ 14, 15, 16, 17 drug induction regimens, in both the frontline and relapsed/refractory settings, as well as in maintenance 18, 19, 20, usually as a single agent. Second primary malignancies have emerged as an issue of significant concern with this agent 21. In general, these tend to be myeloid neoplasms and solid tumors, occurring mostly in the context of melphalan exposure 22.

## Case Report

**Figure 1:**
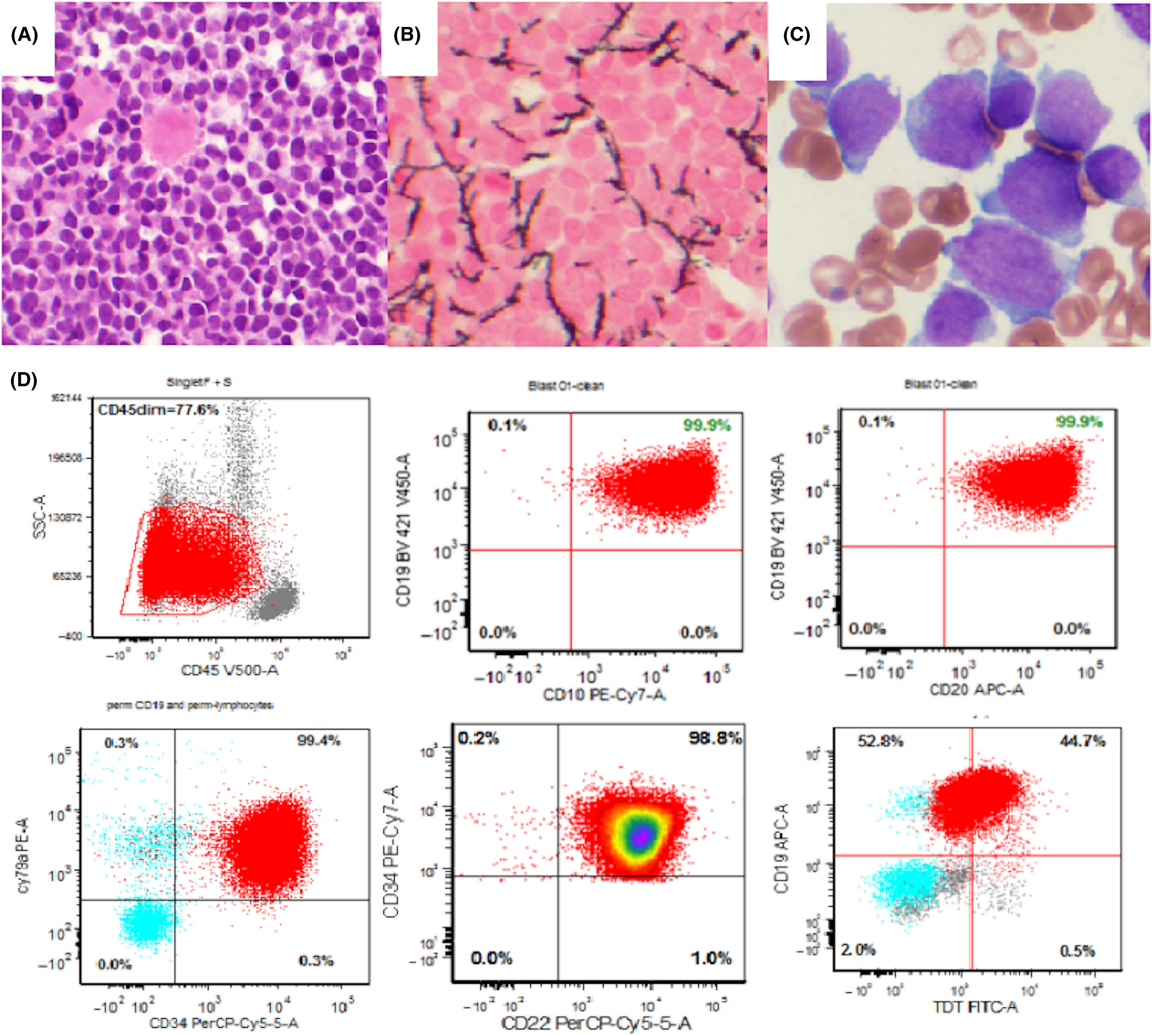
(A) Hypercellular bone marrow (>95%) with heavy infiltration by blasts (88%). (B) Reticulin stain (0–3+) showing focal minimal increase in reticulin fibrosis (1+). (C) Bone marrow smear with markedly increased blasts that were positive on flow cytometry (D) for CD10, CD19, CD20, CD22, and TdT.

A 66‐year‐old Caucasian female with a nearly eleven‐year history of MPN presented to the MD Anderson Cancer Center (MDACC) in January 2016 with a recent diagnosis (December 2015) of B‐ALL. The patient had presented initially with anemia and leukopenia. She had no active infection or bleeding. She also did not have splenomegaly, either by clinical or ultrasonographic evaluation. The diagnosis was confirmed by the finding of 88% blasts in a 95% cellular bone marrow (BM, Fig. 1), all of which expressed the B‐cell antigens CD19, CD20, and CD22. There was no evidence of MPN. Cytogenetics revealed a complex, monosomal karyotype. Targeted next‐generation sequencing using a validated 28‐gene myeloid panel 23 revealed only the *JAK2* V617F mutation. Additionally, testing for calreticulin (*CALR*) mutations was negative. Treatment with chemoimmunotherapy (Cyclophosphamide, Vincristine, dexamethasone, Rituximab, and Inotuzumab Ozogamicin) was begun. Unfortunately, soon after completing her rituximab infusion on day 2 of therapy, the patient developed severe headache, hypertension, and became increasingly obtunded. Imaging disclosed extensive subarachnoid and intraventricular hemorrhage. Comfort measures were adopted given her very poor prognosis in the setting of severe thrombocytopenia, and she passed away the following day. No inotuzumab ozogamicin was administered. Cyclophosphamide and vincristine were the only cytotoxic drugs the patient received.

The patient had been diagnosed with an MPN, variously described as ET or PMF, in January 2005. *JAK2* mutation status at diagnosis was not available. Initial management had been with anagrelide for thrombocytosis, which was changed to lenalidomide and prednisone in January 2012 because of anemia. She responded well to this regimen in terms of symptomatic improvement, platelet count control, and red cell transfusion independence. BM from 2012 was >90% cellular with grade 2–3 reticulin staining, compatible with PMF, and not significantly changed since June 2009. Repeat BM in May 2013 showed apparent improvement in terms of cellularity (50%) and fibrosis grade (focal, grade 1/3). Interestingly, no *JAK2* (or *MPL*) mutation was detected. *CALR* mutations were not tested for at this stage. In March 2014, lenalidomide was discontinued due to symptomatic disease in the form of B symptoms and therapy with ruxolitinib begun, which the patient remained on until her presentation to MDACC.

## Methods

The unique features of this case prompted us to conduct a literature search for published cases of MPN transforming to ALL. In addition, we were intrigued by our patient's prior exposure to lenalidomide and wondered whether the latter might have contributed to her LT in some way. We searched MEDLINE using PubMed in order to identify reports of patients who developed ALL after an MPN, or in the setting of lenalidomide therapy, using the following keywords: “acute lymphoblastic leukemia” AND “myeloproliferative neoplasm” OR “essential thrombocythemia” OR “essential thrombocytosis” OR “polycythemia vera” OR “myelofibrosis”; and “acute lymphoblastic leukemia” AND “lenalidomide” OR “multiple myeloma” OR “myelodysplastic syndrome” OR “5q^‐^ syndrome,” respectively. A total of sixty‐two articles were retrieved by these searches. The titles and abstracts of these articles were reviewed to select thirteen relevant case reports/series. Further, the reference lists of these articles were reviewed in an effort to find additional articles.

## Discussion

Transformation of MPN to ALL is rare, with only seventeen cases reported in the literature (Table 1). Median time to progression to ALL was 10 years (range 2–25). Transformation carried a very poor prognosis, with 80% mortality reported in the published cases. Most ALLs arising in this context had a B‐cell phenotype. Only a few of these are molecularly annotated. Czader and Orazi reported on disease progression of Ph^−^ MPN and chronic myeloid leukemia at the session on LT of MPN at the Society for Hematopathology/European Association for Haematopathology workshop held in Houston, Texas, from October 24 through 26, 2013; two otherwise unpublished cases of progression of Ph^−^ MPN, one ET and one PET‐MF, to *JAK2* ^V617F+^ B‐ALL were described in this article 24. Ohanian et al. from our group reported a case of PPV‐MF that progressed to B‐ALL with a *JAK2* exon 12 mutation 25. Nagai et al. reported on a patient with *JAK2* ^V617F+^ ET who later developed Ph^+^ B‐ALL, but in this case, the *JAK2* V617F mutation was found only in the CD34^+^ hematopoietic stem and progenitor cells (HSPCs) and not in the CD34^+^CD19^+^ Ph^+^ B‐ALL cells, indicating that the two neoplasms were clonally distinct 26.

**Table 1:** Summary of cases of MPN that transformed to ALL reported in the literature

Case no.	Age/gender	MPN subtype	JAK2 status	Cytogenetics (of ALL)	Time to progression (years)	Phenotype	Clinical outcome	Reference
1	61/M	PMF	NR	Aneuploid	5	B cell	Died	37
2	58/F	PPV‐MF	NR	NR	18	Burkitt's	Died	37
3	54/M	PPV‐MF	Exon 12	Diploid	4	B cell	Died	25
4	63/M	PPV‐MF	NR	NR	6	B cell	Died	38
5	53/M	PMF	NR	NR	2	B cell	Died	39
6	74/M	PPV‐MF	NR	NR	6	Null	Died	40
7	42/M	PV	NR	Del 6q, +8	10	Null	Died	41
8	20/M	PV	NR	NR	10	T cell	Died	41
9	68/F	PV	NR	Complex	25	B cell	Died	42
10	76/M	PV	NR	Diploid	16	Common	Died	43
11	54/F	PV	NR	NR	13	Common	Alive	44
12	65/M	ET	V617F	Del 9p13	16	B cell	Unknown	24
13	59/M	PET‐MF	V617F	Del 13q and 20q	10	B cell	Unknown	24
14	67/F	ET	V617F^a^	t(9;22)	16	B cell	Died	26
15	65/F	ET	Neg^b^	Hyperdiploid	3.5	B cell	Alive	45
16	70/F	ET	NR	Diploid	19	B cell	Unknown	46
17	56/M	PMF	Neg	t(9;22), del 20q	1	B cell	Died	47
18	65/F	PMF	V617F	Complex, monosomal	11	B cell	Died	Present case

NR, not reported; PMF, primary myelofibrosis; PPV‐MF, postpolycythemia vera myelofibrosis; PET‐MF, postessential thrombocythemia myelofibrosis; ET, essential thrombocythemia.

a Not in the ALL clone at transformation.

b *CALR* mutant.

Delhommeau and colleagues studied the presence of the *JAK2* V617F mutation in circulating B‐, T‐, and natural killer (NK) cells of patients with PV and PMF, and detected it in B‐ and NK cells from approximately half their patients with PMF and a minority of those with PV 27. Furthermore, a few patients with PMF also carried the mutation in their peripheral T cells. The mutation was subsequently detected in B/NK/myeloid precursors from PV and PMF patients, as well as in T‐cell fractions derived from CD34^+^ cells, thus showing that MPN originates in a true myeloid/lymphoid progenitor cell, although the proliferative advantage conferred by the driver mutation appears to be limited to the myeloid lineage 27 **.** This would be consistent with the two‐hit theory of leukemogenesis, suggesting that other genetic lesions are also involved in MPN pathogenesis. Indeed, it has been suggested that *JAK2* ^V617F^ only confers a weak proliferative advantage on the hematopoietic stem cell (HSC), such that on its own, it would cause an MPN with a very long latency; thus, *JAK2* ^V617F^‐bearing HSCs may remain harmless for a long time, until genetic or environmental changes such as hematopoietic stress or aging allow clonal dominance and MPN emergence 6.

Only eight cases of ALL, all with a B‐cell phenotype, arising as a second malignancy in lenalidomide‐treated patients, have been reported in the literature. Median duration of lenalidomide therapy in the reported cases (Table 2) was 3 years (range 2–7). Most patients were receiving the drug for MM; two cases were reported in patients who were on the immunomodulatory agent for MDS with a 5q deletion 28. Different doses and schedules were reported, and the patients with MM received lenalidomide during induction or as maintenance therapy, or both (Table 2). In contrast, the increased risk of myeloid neoplasms and solid tumors has been well described in the setting of prolonged lenalidomide maintenance therapy of MM, mainly in the context of prior exposure to high‐dose melphalan conditioning 22. Indeed, in the three large randomized controlled trials evaluating maintenance therapy with lenalidomide in a total of 690 patients with MM, only five cases of ALL were reported, four of them in the post‐transplant maintenance setting 18, 19, 20.

**Table 2:** Summary of cases of ALL reported in the setting of lenalidomide therapy

Case no	Age/gender	Diagnosis	Timing	Dose/schedule	Duration of therapy (years)	Phenotype	Clinical outcome	Reference
1	59/M	MM	Induction & Maintenance	NR	2.5	B cell	Alive	48
2	34/M	Rel. MM	Induction & Maintenance	5 mg/TIW	3	B cell	Died	48
3	53/M	MM	Induction & Maintenance	25 mg/daily	7	B cell	Alive	48
4	52/F	AL	Induction	15 mg/daily 21/28	4.5	B cell	Died	49
5	72/M	MM	Maintenance	NR	3	B cell	Unknown	50
6	62/F	MM	Induction & maintenance	10 mg/daily 21/28	2	B cell	Died	51
7	68/M	MDS 5q‐	Induction	NR	2.5	B cell	Died	28
8	83/F	MDS 5q‐	Induction	5 mg/daily	6	B cell	Died	28
9	65/F	PMF	N/A	10 mg/daily	2	B cell	Died	Present case^a^

MM, multiple myeloma; MDS, myelodysplastic syndrome; NR, not reported; AL, light chain amyloidosis; N/A, not applicable; TIW, three times a week.

a Patient was off lenalidomide when transformation occurred.

The mechanisms of action of lenalidomide in MM 29 and MDS with del5q 30, involving the cereblon‐dependent destruction of Ikaros family transcription factors and casein kinase 1A1, respectively, have only recently been elucidated, and how the drug may affect an MPN clone over time remains largely unknown. Although not specifically approved for this indication, lenalidomide, with or without prednisone, is often used for the treatment of myelofibrosis 31, 32, 33, particularly for the amelioration of anemia, which does not usually improve significantly with ruxolitinib. However, simultaneous administration of lenalidomide and ruxolitinib is difficult 34. Some experts reserve lenalidomide for patients with del5q given the potential for myelosuppression and thrombosis with this agent 35, although this particular cytogenetic abnormality is extremely rare in myelofibrosis 36.

## Conclusion

Our case illustrates the pluripotency of the JAK2^V617F+^ stem/progenitor cell, as do a few others reported in the literature. The incidence of ALL occurring in the context of lenalidomide therapy is very low, precluding definitive conclusions.

## Authorship

AA and PB wrote the manuscript and AA performed literature searches. PB and SV reviewed the paper for important intellectual content. AA, PB, KN and CH provided clinical care to the patient. YOH performed hematopathologic evaluation of the cases and provided the bone marrow morphology and flow cytometry images.

## Conflict of Interest

None declared.
